# Effects of Parathyroid Hormone on Calcium Ions in Rat Bone Marrow Mesenchymal Stem Cells

**DOI:** 10.1155/2014/258409

**Published:** 2014-06-18

**Authors:** Yushu Chen, Bo Bai, Shujiang Zhang, Jing Ye, Yi Chen, Yanjun Zeng

**Affiliations:** ^1^Department of Orthopaedic Surgery, Orthopedics Implantation Key Lab of Guangdong Province, 1st Affiliated Hospital of Guangzhou Medical University, No. 151 Yanjiang Road, Guangzhou 510120, China; ^2^Department of Orthopaedic Surgery, 1st People Hospital of Yunfu, No. 100 Construction of North Road, Yunfu 527300, China; ^3^Biomechanics & Medical Information Institute, Beijing University of Technology, No. 100 Flat Land Chaoyang District, Beijing 100022, China

## Abstract

The present study was conducted in order to explore the mechanisms whereby parathyroid hormone (PTH) maintains *in vitro* proliferation of bone marrow mesenchymal stem cells (BMSCs). Bone marrow was isolated from Sprague Dawley (SD) rat femurs, cultured *in vitro*, and passaged using a cell adherent culture method. The BMSC proliferation was evaluated by the methyl thiazolyl tetrazolium (MTT) assay and the fluorescence intensity of calcium ions in BMSCs was analyzed by laser scanning confocal microscopy (LSCM). Our results show that BMSC proliferation in the experimental group treated with PTH was more significant than controls. The calcium ion fluorescence intensity in BMSCs was significantly higher for the experimental group as compared to the control group. For each group, there was significant difference in the fluorescence intensity of calcium ions in BMSCs between 7 d and 14 d. In conclusion, parathyroid hormone increased the fluorescence intensity of calcium ions in BMSCs, which might represent a key mechanism whereby BMSC proliferation is maintained.

## 1. Introduction

Bone marrow mesenchymal stem cells (BMSCs) are found in the bone marrow and belong to stem cells that display a potential for multilineage differentiation. BMSCs have been a key focus in stem cell research. The frequency of BMSCs is relatively low in the bone marrow and accounts for approximately 0.001%–0.1% of the total nucleated cells [1–3]. Hence, it is crucial to maintain BMSC cell proliferation* in vitro*. Recent studies have shown that intermittent PTH administration could increase the BMSCs differentiation into the osteoblast and improve its activity [4, 5]. Simultaneously some investigators have suggested that PTH treatment may affect the number of hematopoietic stem cells in the bone marrow and their mobilization into the bloodstream. Cells with classical features of mesenchymal stem cells/progenitors have been shown to express receptors for PTH, increase in number, and undergo redistribution in the adult bone marrow upon PTH treatment [6]. Furthermore, Di Bernardo et al. also indicated that PTH could strengthen the proliferation rate of MSCs with a diminution of senescence and apoptosis [7]. However, the regulation mechanisms of parathyroid hormone (PTH) in maintaining* in vitro* proliferation of bone marrow mesenchymal stem cells (BMSCs) are still not clear at present. Calcium ions (Ca^2+^) are appreciated as the second most important messengers in cells and play a unique role in the maintenance of normal physiological activities [8]. The content of Ca^2+^ in cells changes and can directly affect cell proliferation, differentiation, and signal transduction [8].

Parathyroid hormone (PTH) is a polypeptide hormone synthesized and secreted by chief cells of the parathyroid gland. PTH is also a G protein-coupled receptor signaling protein with catabolic and anabolic functions. PTH can also regulate physiological calcium balance by activating a series of physiological and biochemical reactions by a specific receptor that is displayed on the surface of its target organ [9, 10].

In this study, we investigated the effects of PTH on Ca^2+^ fluorescence intensity of BMSCs following* in vitro* culture at different culture times using laser scanning confocal microscopy (LSCM) and Fluo-3/AM specific fluorescent staining. Using these technological approaches, we explored related mechanisms and provided valuable information for studies on the application of BMSCs in tissue engineering.

## 2. Materials and Methods

### 2.1. SD Rats

Ten one-week-old SD rats (male and female) were provided by the Animal Experiment Center of Guangdong Academy of Medical Sciences, China (animal license number: SCXK Guangdong 2008-0002).

### 2.2. BMSC Isolation, Culture, and Identification

SD rats were euthanized and then immediately immersed in povidone-iodine for 10 mins. Rat femurs were isolated from the demised SD rats under a sterile biological Class II-B safety cabinet (Altai Laboratory Equipment Co., Ltd.). The soft tissue surrounding the femurs was removed; following that, they were rinsed in phosphate buffered saline (PBS, Institute of Biomedical Engineering, The Chinese Academy of Medical Sciences, China) containing penicillin and streptomycin for a total of three washes. After the epiphyseal region was dissected away, the marrow cavity was exposed and repeatedly washed with 1 mL syringes filled with L-DMEM (Gibco, USA) medium containing 15% fetal bovine serum (FBS, Hyclone). The whole volume of the flushing fluid was added to the single cell suspension, incubated in 25 cm^2^ plastic culture flasks, supplemented with 3 mL L-DMEM medium containing 15% FBS, and cultured in an incubator (Thermo, USA) at 37°C and 5% CO_2_ in air and saturated humidity. The culture medium was changed every 2-3 d. The culture was ended when 80% of the cells completed trypsin digestion and passage. The study complied with the Guidance of Experimental Animals Treatment [11]. BMSCs of passage four were collected and prepared as cell suspensions. The CD44 (LifeSpan Biosciences, St. Louis, USA) expression in suspensions was detected by flow cytometry, cultured in osteoblast-inducing conditioned media, and then analyzed by the alkaline phosphatase staining method [12].

### 2.3. Methyl Thiazolyl Tetrazolium (MTT) Assay of BMSC Proliferation

Passage 4 BMSCs were collected and prepared as cell suspensions (2 × 10^4^/mL). The suspensions (100 *μ*L) were added to each well of a 96-well plate. The wells were randomly divided into three groups. One group (the experimental group) was added in L-DMEM containing 10 nM PTH (1–34) (Biovision, USA), the other group (the suppression group) was added in L-DMEM containing 10 nM PTHrP (7–34) (Biovision, USA), and the third group (the control group) was added in L-DMEM without PTH. During the incubation, the culture medium was replaced every three days. On the day of assay, each well was added with 10 uL of MTT (5 mg/mL) (Sigma) and incubated at 37°C. After 4 hrs, the supernatant was discarded and each well was exposed to 150 uL of dimethyl sulfoxide (DMSO) to solubilize the formazan product. After 10 min oscillation, the optical density of each sample was determined at a wavelength of 490 nm (A_490_) by microtiter plate spectrophotometry. The growth curves of BMSCs in each group (A_490_ versus time (d)) were then plotted.

### 2.4. LSCM Method Detecting Ca^2+^ Fluorescent Intensity in BMSCs

BMSCs at passage 4 were collected and prepared as single cell suspensions (1 × 10^4^/mL). Cell suspensions (1 mL) were inoculated onto four confocal dishes (NEST Biological Technology Co., Ltd., Shanghai, China). The dishes were randomly divided into two groups. One group (the experimental group) was cultured in L-DMEM containing 10 nM PTH, and the other group (the control group) was cultured with L-DMEM in the absence of PTH. During the incubation the culture medium was replaced every three days. At 7 d and 14 d after culture, the intensity of the calcium fluorescence was assayed and cell morphology was determined.

BMSC specimens were washed in calcium- and magnesium-free PBS buffer (pH7.2) three times and labeled with 10 *μ*mol/L Fluo-3/AM (Biorad, USA) at 37°C. After 30 min, BMSC specimens were washed in PBS three times to remove BMSC extracellular fluorescent dye. Then, each dish was incubated with 1 mL H-DMEM medium (Gibco). After equilibration, Ca^2+^ fluorescence intensity and BMSC cell morphology in each dish were detected by LSCM (Carl Zeiss, Germany) with an excitation wavelength of 488 nm and an emission wavelength of 526 nm at 30°C.

## 3. Statistical Analysis

All data are expressed as mean ± standard deviation (SD). SPASS version 16.0 (SPSS Inc., Chicago, IL, USA) was used to perform analysis of variance (for repeated measures) for all inter- or intragroup comparisons. For all analyses, a probability less than an alpha value of 0.001 (*P* < 0.001) was considered statistically significant.

## 4. Results

### 4.1. BMSCs Growth

**Figure 1:**
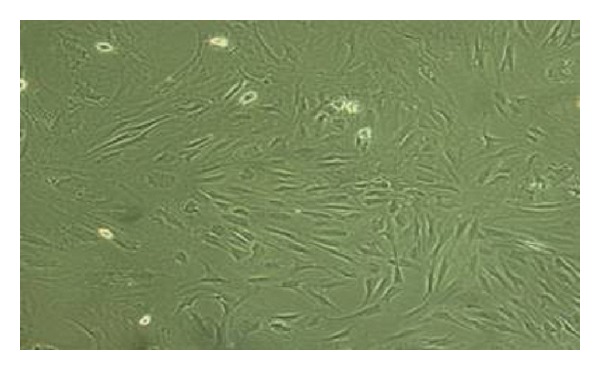
Bone marrow mesenchymal stem cells at passage number four (×100 magnification).

In 48 hrs after BMSCs were cultured onto 96-well plates, spindle-shaped or polygonal adherent cells were found. On the third day, the number of adherent cells increased. Cells had polygonal protuberances and grew in a fibroblast-like colony. On the tenth day, cell fusion reached 90%, and cells were tightly packed in swirling- or radial-like shapes, indicating that cell passages were possible. Cell passages were performed every 5–7 days. BMSCs were obtained with higher purity for each passage. The BMSCs of passage 4 were of the highest purity (Figure 1). After passaging, adherent cells grew rapidly.

### 4.2. BMSCs Identification

**Figure 2:**
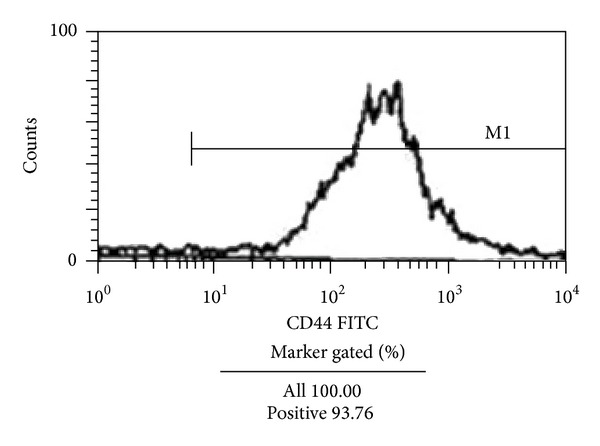
Expression of cell surface markers in cultured BMSCs as detected by flow cytometry. The surface marker CD44 was positively expressed by 93.76% of the cells.

**Figure 3:**
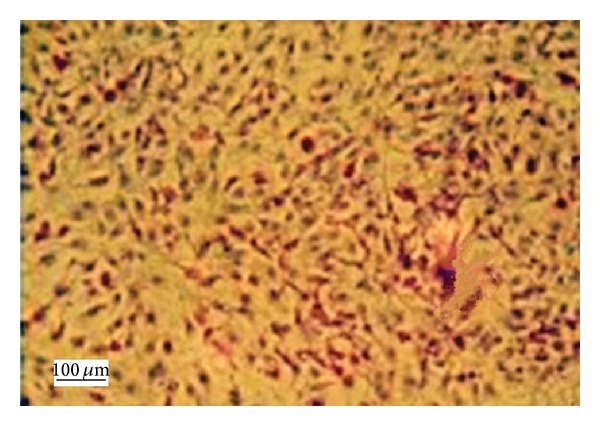
Osteogenic differentiations of BMSCs (×100 magnification). Dark brown particles represent positive results and were observed after osteogenic-induced BMSCs were stained with the alkaline phosphatase procedure.

Flow cytometric analysis revealed that CD44 expression in BMSCs at passage 4 was highly expressed with a positive rate of 97.36% (Figure 2). After osteoblast-induced BMSCs at passage 4 were stained with alkaline phosphatase, brownish red particles were found in the cytoplasm, and alkaline phosphatase was positively expressed (Figure 3).

### 4.3. BMSCs Proliferation

**Figure 4:**
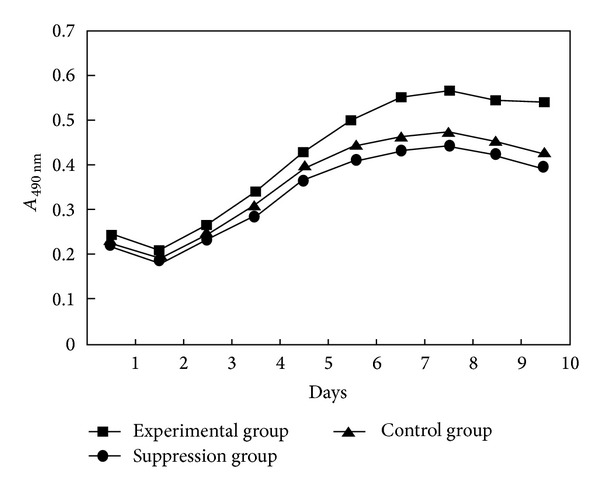
The growth curve of BMSCs in the experimental, suppression, and control groups (Δ*P* < 0.001 between experimental group versus suppression group and control group; *P > 0.001 between suppression group and control group).

BMSC proliferation was analyzed by cell growth curve determination (Figure 4). After 1-2 days of culture, cell growth in the experimental, suppression, and control groups was slow. However, after 2-3 days of culture, cells grew rapidly. After 7-8 days, cell growth reached peak activity. On the tenth day, cell proliferation started to decrease and this decrease in cell proliferation was more significant in both the suppression group and the control group than in the experimental groups (*P* < 0.001), whereas the cell proliferation in the suppression group and the control group was not of a statistically significant difference (*P* > 0.001).

### 4.4. Ca^2+^ Fluorescence Intensity in BMSCs

**Figure 5:**
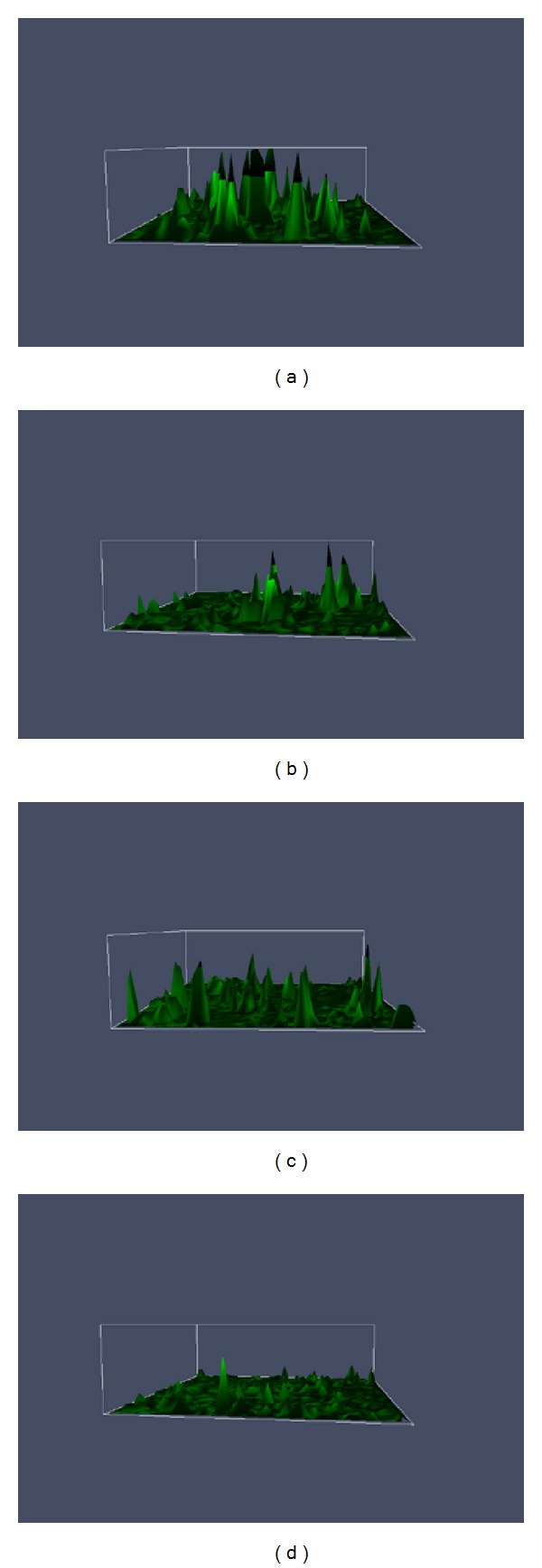
Two-dimensional intracellular calcium fluorescence spectra. (a) The experimental group (on the 7th day); (b) the experimental group (on the 14th day); (c) the control group (on the 7th day); (d) the control group (on the 14th day).

**Figure 6:**
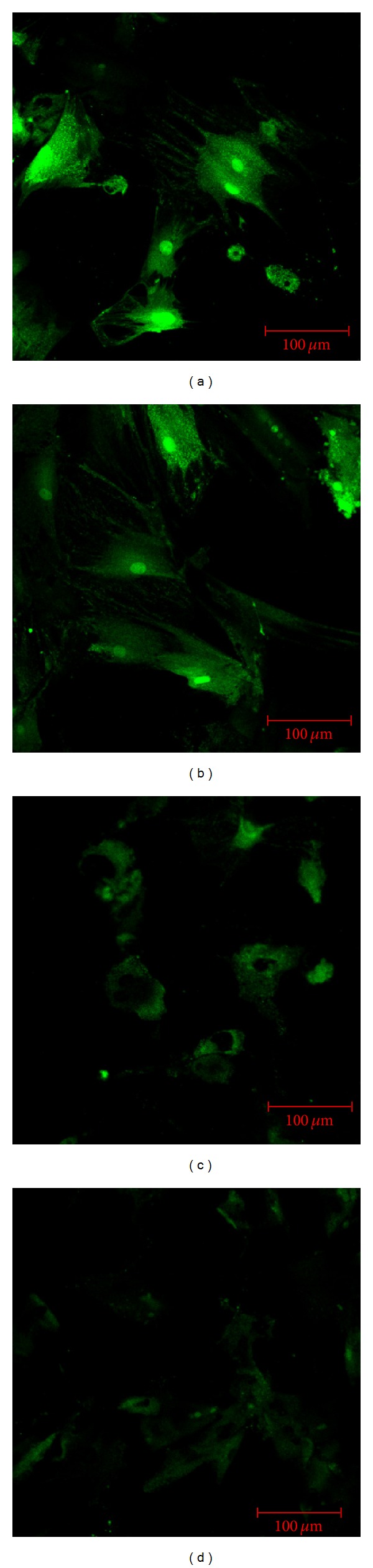
Fluorescence spectra of BMSCs on day 7 (a) and day 14 (b) in the experimental group, as compared to day 7 (c) and day 14 (d) in the control group. (a) BMSCs were polygonal with strong green fluorescence in the cytoplasm. (b) Polygonal BMSCs had decreased in number and cytoplasmic green fluorescence was dampened in the experimental group by day 14 as compared to day 7. (c) BMSCs became spherical or elliptical, and the cytoplasmic green fluorescence was markedly weaker in the control group than in the experimental group on day 7. (d) There were no obvious polygonal BMSCs, and the cytoplasmic green fluorescence was the weakest among the groups.

On the seventh day following culture, high Ca^2+^ fluorescence intensity was found in BMSCs of both the experimental and control groups. On the 14th day, Ca^2+^ fluorescence intensity of BMSCs was significantly (*P* < 0.001) decreased in both groups. At each culture time, the Ca^2+^ fluorescence intensity of BMSCs was significantly (*P* < 0.001) higher in the experimental group than that found in the control group. The Ca^2+^ fluorescence intensity of BMSCs in the experimental group on the 14th day of culture was not significantly (*P* > 0.001) different from that in the control group on the 7th day (see Table 1 and Figure 5). Cell morphology was significantly more intact in the experimental group than that found in the control group (Figure 6).

## 5. Discussion

BMSCs are found in a variety of organs and tissues but are most abundant in the bone marrow. Hence, BMSCs from the bone marrow are widely studied [13]. The content of bone marrow BMSCs is low (about one BMSC in 100,000 nucleated cells), and it diminishes with increasing age [1, 14]. In our* in vitro* environments, only a small proportion of BMSCs proliferated. Therefore, to find methods that isolate, purify, and amplify BMSCs* in vitro* is necessary to maintain BMSC proliferation and forms a basis for further BMSC study. In recent years, methods used to isolate and purify BMSCs are mostly dependent on enriching whole bone marrow cells by a plastic substrate adherent method, density gradient centrifugation techniques, flow cytometric separation tools, and immunomagnetic bead cell separation technology. The density gradient centrifugation technique, flow cytometric separation, and immunomagnetic bead cell separation technology can efficiently screen BMSC with a high degree of purity but can also change the external microenvironment of BMSCs, which can affect cell viability and cause cell loss [15–17].

The whole bone marrow cell adherent method isolates cells from nonadherent cells and other impurities based on characteristics of BMSC adhesion to the plastic substrate of the culture flasks. It is a relatively simple, robust, and reliable method and can produce a higher purity of BMSCs by changing the culture medium and cell passage [1]. This study used the whole bone marrow cell culture method for the separation and purification of BMSCs. We obtained BMSCs with the highest purity possible when cells were passaged to the fourth generation. Meanwhile, cells were in spindle or polygonal shape and grew in good condition.

BMSCs are able to differentiate into pluripotent stem cells such as muscle cells, fat cells, chondrocytes, and osteoblasts and can express different cell surface markers [1, 18]. Additionally, BMSCs can be regulated by PTH and are widely used in tissue engineering [1, 18]. With the development of tissue engineering, the demand for seed BMSCs is growing. Thus, how to maintain BMSC proliferation has become an important clinical research consideration. It was reported that PTH significantly increases the proliferation rate of BMSCs, reduces their senescence and apoptosis, and maintains the integrity of its genome [7, 19–21]. However, it is unclear how the mechanisms of PTH promote the proliferation of BMSCs.

Intracellular Ca^2+^ is one of the most important second messengers and is essential for life activity. Changes in the concentration of Ca^2+^ cations in cells can directly affect cell proliferation, differentiation, and signal transduction [8]. Therefore, it is meaningful to monitor changes in Ca^2+^ in cells.

In recent years, confocal microscopic technology in conjunction with a new generation of fluorescent dyes has been widely used for dynamic changes analysis of calcium ions in living cells. The fluorescent indicator Fluo-3/AM is fat-soluble and can enter cells across the plasma membrane. After hydrolysis by nonspecific esterases in living cells, Fluo-3/AM can highly specifically combine with intracellular free calcium and generate fluorescence if excited by light of a certain wavelength. Fluo-3/AM is a laser probe with a single wavelength, and its fluorescence intensity is proportional to the density of Ca^2+^ cations, and hence it can accurately reflect changes in calcium concentration [22].

In this study, the fluorescent indicator Fluo-3/AM was used to monitor calcium fluorescence intensity in cultured BMSCs, and the results showed that calcium fluorescence intensity of BMSCs that were treated with PTH in the experimental group was comparably and significantly higher than that in the control group.

Under normal circumstances, intracellular free Ca^2+^ concentrations may change, and cyclical changes reflect the physiological functions or stresses of cells. When stimulated by external factors, the concentration of intracellular Ca^2+^ correspondingly changes. The fluorescence intensity of Ca^2+^ can indirectly reflect the response of Ca^2+^ to stimuli and its related biological effects. Thus, monitoring the fluorescence intensity of Ca^2+^ can be used to explore molecular mechanisms of biological effects caused by external stimuli or danger signals [23]. The effects of calcium in cells stimulated by media are regulated by extracellular calcium-sensing receptors (CaSRs), which are G protein-coupled receptors. The receptors can be expressed in a variety of tissues and can activate the protein kinase-signaling cascade. Therefore, CaSR can activate extracellular signal-regulated kinase phosphorylation via protein kinase signaling pathways and maintain cell proliferation for a prolonged period [24, 25]. Parathyroid hormone is also a G protein-coupled receptor signaling protein and can regulate calcium balance by activating a series of physiological and biochemical reactions by specific receptors that are expressed on the surface of target organs and results in calcium effects [9, 10]. This study found that calcium fluorescence intensity in BMSCs was significantly higher in the experimental group (PTH treatment) than that found in the control group at the same culture times.

Meanwhile, the morphology of BMSCs in the experimental group was much improved as compared with that in the control group. The effects of PTH on BMSCs were weakened in accord with increasing culture time. However, BMSCs in the experimental group retained good cell morphology. The Ca^2+^ fluorescence intensity of BMSCs in the experimental group on the 14th day of culture was not significantly (*P* > 0.001) different from that in the control group on the 7th day of culture. The cell growth curves also revealed that BMSC proliferation was significantly higher in the experimental group following PTH treatment as compared to the control group. This observation indicated that PTH may lead to calcium effects, at least in some way, which may represent a formal mechanism, whereby PTH is capable of maintaining BMSC proliferation.

## Figures and Tables

**Table 1:** Calcium ion fluorescence intensity of BMSCs in the experimental and control group.

Group	*n*	Calcium ion fluorescence intensity	
		7 d	14 d
Experimental group	6	7.91 ± 2.34^∗^	5.15 ± 1.58^∗^^∗^
Control group	6	5.37 ± 2.55^∗^^∗^	2.71 ± 0.89^∗^

**P < 0.001 between two sampling times, or between groups.

*P > 0.001 between experimental group on the 14th day and control group on the 7th day.
